# Characteristics of Collective Resilience and Its Influencing Factors from the Perspective of Psychological Emotion: A Case Study of COVID-19 in China

**DOI:** 10.3390/ijerph192214958

**Published:** 2022-11-14

**Authors:** Siyao Liu, Bin Yu, Chan Xu, Min Zhao, Jing Guo

**Affiliations:** 1 College of Urban and Environmental Sciences, Central China Normal University, Wuhan 430079, China; 2 Key Laboratory for Geographical Process Analysis & Simulation of Hubei Province, Central China Normal University, Wuhan 430079, China; 3 The Faculty of Geography & Resource Sciences, Sichuan Normal University, Chengdu 610101, China; 4 Key Laboratory of the Evaluation and Monitoring of Southwest Land Resources, Ministry of Education, Sichuan Normal University, Chengdu 610068, China

**Keywords:** psycho-emotional, collective resilience, grounded theory, sentiment analysis, COVID-19, China

## Abstract

Collective resilience is the ability of human beings to adapt and collectively cope with crises in adversity. Emotional expression is the core element with which to characterize the psychological dimension of collective resilience. This research proposed a stage model of collective resilience based on the temporal evolution of the public opinions of COVID-19 in China’s first anti-pandemic cycle; using data from hot searches and commentaries on Sina Weibo, the changes in the emotional patterns of social groups are revealed through analyses of the sentiments expressed in texts. A grounded theory approach is used to elucidate the factors influencing collective resilience. The research results show that collective resilience during the pandemic exhibited an evolutionary process that could be termed, “preparation–process–recovery”. Analyses of expressed sentiments reveal an evolutionary pattern of “positive emotion prevailing–negative emotion appearing–positive emotion recovering Collective resilience from a psycho-emotional perspective is the result of “basic cognition-intermediary condition-consequence” positive feedback, in which the basic cognition is expressed as will embeddedness and the intermediary conditions include the subject behavior and any associated derived behavioral characteristics and spiritual connotation. These results are significant both theoretically and practically with regard to the reconstruction of collective resilience when s‘ force majeure’ event occur.

## 1. Introduction

As the world today continues to evolve both irrationally and inequitably, human society is entering what we may term an “eventful period”. There are frequent disasters, rampant viruses, rife disputes, and various uncertain risks which pose a serious threat to human survival. The concept of resilience, which is rooted in psychology as an enquiry into individual persistence, can now be viewed as converging with explorations into the dynamics of the ecosystem. It has grown into a broad and exciting field of study, gaining popularity in the academic sector, as well as capturing the interest of policy makers and the general public. Not only does resilience thinking provide an increasingly vigorous and sophisticated body of analysis, it also offers the prospects of more integrated and effective policy-making in a drive towards sustainability [1]. Or, to some extent, “resilience is to the 2000s and 2010s what sustainability was to the 1980s and 1990s” [2]. For these reasons, building resilience has become an inevitable choice for coping with shared risks and challenges, as well as for informing future sustainable development. Generally, resilience includes both physical resilience and social resilience which can be further divided into individual resilience and collective resilience. The former refers to a group of attributes possessed by an individual, that enables that individual to have strength and perseverance when facing major setbacks and challenges in life. The latter refers to the way people in crowds express and expect solidarity and thereby coordinate and draw upon collective sources of practical and emotional support to adaptively deal with an emergency or disaster [3]. In this account, the collective resilience of unstructured crowds within emergencies should be deemed a psychosocial resource, and is therefore treated as the cornerstone of any post-disaster recovery [4]. This psychosocial resource arguably consists of four dimensions, i.e., structure, psychology, capital and environment. The psychological dimension represents collective behavior and decision making, both of which are influenced by psychological perceptions and motivations, and both of which thus play a crucial role in forming collective resilience [5].

Positive emotions are one of the key components of the psychological dimension of collective resilience. When there is a crisis, a positive attitude can be a functional manifestation of psychological resilience [6]. It has also been demonstrated by the ‘broaden-and-build’ theory that positive emotions may not only reflect and fuel psychological resilience, but that they might also build psychological resilience over time [7]. Emotions are important determinants in risk perception, a key predictor of the motivation required to take protective action as well as the subsequent behavioral performance aimed at alleviating any threat. Of the three components that comprise risk perception (i.e., the deliberative, affective and experiential components), the affective component emerges as the strongest predictor to shape the reaction of groups to risky events, and effectively change group behavior [8,9].

In addition, some social psychologists have integrated appraisal theories, social identity theory, and self-categorization theory into the Intergroup Emotions Theory, IET, which interprets the social and intergroup regulatory functions of group emotions. These scholars have argued that group emotions provide the behavioral motivations, desires or inclinations required to facilitate or alienate collective relationships and identity, change or reinforce status hierarchies, eliminate competitors, and build alliances [10].

This research argues that the psychological adaptation and recovery of the group, that is, collective psychological resilience, is an invaluable factor in responding to unexpected public crises, such as the outbreak of COVID-19. When it comes to identifying and characterizing collective resilience in public crises, psychological emotions can be a valuable lens through which the global structure and detailed regional patterns can be well presented. In view of this, this article uses text review sentiment analysis and grounded theory to explore the nature of collective resilience during the COVID-19 pandemic in China. It attempts to discuss the evolution of public opinion and emotional expressions about mass focus events in the context of the pandemic, reveal the characteristics of collective resilience development processes and changes in emotional behaviors during an emergent public crisis, and to construct an influencing factors model of collective resilience from a psycho-emotional perspective. The hope is that this may, offer an empirical model of collective resilience that could allow society to cope better with future crise and risks.

## 2. Literature Review

Psychology has long been interested in how people resume healthy functioning after suffering from traumatic experiences, such as wars, accidents, or life-threatening diseases [11]. Resilience in psychology is defined as “good outcomes in spite of serious threats to adaptation or development” [12] or “the ability to recover from or adjust easily to misfortune or sustained life stress” [13]. Although the use of resilience in psychology began early in the 1950s, it did not become an active area of research in this field until the late 1980s [14]. In recent years, psychologists and sociologists have explored the psychological dimension of collective resilience in the context of social inequality, conflict or external shocks. The existing research was primarily focused on the factors and mechanisms of collective resilience. First and foremost, social and local identity, a sense of collectivism, a feeling of belonging, and a collective cultural identity are recognized as the main factors affecting psychological resilience.

The positive impact of group social identities, such as common fate and collective memory, on building psycho-social resilience in the aftermath of natural disasters has been well demonstrated [15,16]. Black women who were exposed to human rights deprivation could enhance their psychological resilience primarily through improving access to community resources, promoting a sense of group belonging, and expanding public services in health and education [17]. For refugees, the recovery of collective cultural, religious and ethnic identity after migration directly correlated with their psychological well-being [18]. Children caught up in war have been able to reduce their emotional distress resulting fromcollective violence using self-regulation and parental and social support, thus bolstering their psychological resilience [19]. A decline in racial prejudice and a strengthening of ethnic identity has been crucial for the psychological well-being of Asian American communities as they have struggled to overcome both the pandemic and racism [20].

The other side of the coin has been the research into the role and influence mechanisms of group psycho-emotion. Psychological manifestations of collective resilience included emotional identification within the groups, attitudinal and behavioral convergence, as well as positive social responses. With the proliferation of public opinion about social events, groups shared and exchanged emotions through places to foster a sense of community and social solidarity, as well as to promote behavior convergence among groups, thus generating collective identity, achieving common beliefs, and realizing positive social responses. During this process, group emotions could be automatically and rapidly constructed and experienced [21,22,23]. On the other hand, positive emotion can boost collective resilience, contributing to the formation of collective value orientations, alleviating group suffering during disasters, and thus decreasing the negative impact of collective crises [24]. Furthermore, the rapid growth of a networking society and the rise of big data in particular have led academics to consider online groups as important research resource banks that can allow them to explore public psychological resilience as represented by the emotions expressed within them [25,26]. Netizens have formed a unique socio-cultural collective identity in online socialization, which can break down the barriers between possible cultural communication, as well as enable the decolonization process to occur and building the capacity for collective resilience [27].

Resilience studies related to COVID-19 have proliferated in a short time following the outbreak. The pandemic has illustrated the remarkable collective resilience of individuals coming together as a community through the common experience of crisis. The factors commonly recognized as having had an impact on collective resilience in the face of the pandemic include collective efficacy, social leadership and fairness, racial or ethnic identity, policies and cultural forms, faith and social connection, flexibility, and social cohesion [20,28,29,30,31,32]. Social cohesion includes trust, a sense of belonging, social interactions and engagement [33]. The huge impact of COVID-19 required considerable adaptations of healthcare workers. Scholars have therefore focused their research on this particular group, suggesting that engaging staff at every stage, creating a sense of connection with others, collective humanistic care, social support, team unity, and realizing their self-worth with a sense of sacred mission all contributed to the adaptation and recovery of the healthcare community during the pandemic [34,35]. However, fragmented guidance and communication, a sense of unfairness generated by relational challenges, uncertainty over safety, and variable referral quality caused them to feel undervalued and frustrated, thereby exerting a negative impact on their psychological adaptation and recovery [36]. The complex interplay between emotions and resilience has also been discussed in depth. Positive emotion can help people trapped in adversity cultivate a resilient mindset [37,38], and its regulation of such emotions is important for maintaining a group’s mental health [39,40]. Collective resilience has also been identified as one of the determinants of optimism [41].

To summarize, the exploration of collective resilience is principally based on qualitative methodologies. There is a lack of empirical research into quantitative factors. There is a relatively limited amount of empirical research into collective resilience during public disasters, and it remains to be discovered exactly what roles the psychological and emotional aspects of such disasters have played over the long term. This paper posits the view that social groups represent collectively shared resources, and that collective resilience can play a unique role in the aftermath of public disasters as well as in any post-disaster psychological reconstruction process. The COVID-19 pandemic in China will be used as an illustration in this study to examine the traits and determinants of collective resilience from a psycho-emotional point of view.

## 3. Data and Methods 

### 3.1. Overview

With the advent of the mobile internet era, Sina Weibo (hereinafter referred to as Weibo), one of the earliest and most used social media platforms in China, has rapidly expanded its user base and increased its user activity, making it an information access platform with extensive influence. In particular, during the first outbreak of the COVID-19 pandemic in mainland China from late 2019 to early 2020, hundreds of millions of users followed the latest progress of the pandemic through Weibo, including the number of confirmed cases, protective measures, and material donations. Weibo not only became the main battlefield of information dissemination, but also the largest platform for the public to express their opinions and release their emotions after extensive lockdowns imposed during the pandemic. Since the outbreak of COVID-19, the extraction of the semantic or emotional content of textual commentaries posted by the public has become a useful research tool to explore the evolution of topics and the emotional expression characteristics of particular groups Due to the prolonged series of COVID-19 lockdowns, social media has also become a placeholder for collective resilient processes modulated by cognitive and emotional components [42]. This article also draws on a similar research approach, using Weibo data and Baidu indexes, to investigate the collective resilience characteristics of social groups during an emergent public crisis, taking randomly-selected unstructured groups with different social attributesfrom the Weibo platform as research subjects [25,43,44,45,46,47,48,49].

On 9 January 2020, the phrase “Wuhan unexplained pneumonia pathogen is a new type of coronavirus” was on the hot search list, and the pandemic started to attract social attention. With a huge governmental and societal efforts the rapid rise of the pandemic was contained. After 21 February, provincial public health response levels were successivelylowered and traffic restrictions were gradually canceled. The resumption of work and production was resumed under the “Guidelines on Pandemic Prevention and Control Measures for Resumption of Work and Production for Enterprises and Institutions” issued by the State Council. The research argues that the 9 January 2020 to 21 February 2020 period witnessed a complete cycle in China, from the confirmation of COVID-19, to the spread of the pandemic, to the basic control of the virus. This was China’s first anti-pandemic cycle; it therefore typifies a group fight against the pandemic. We therefore chose the 9 January to 21 February 2020 window as our research period.

### 3.2. Data Collection and Processing

According to the Weibo Users Development Report 2020, 12:00 h is the prime time for the public to log on to the internet, i.e., this is the time when Weibo has the largest number of online users. Since the Weibo platform can only provide users with 50 hot search terms at any one time, the Python tool was used to extract the top 50 hot searches on Weibo at 12:00 h each day and then data cleaning was conducted. Considering the uniqueness of the pandemic-related topics required in this article and the complexity of pandemic events in China at that time, as well as the diversity of Chinese expressions about the pandemic, non-manual operations such as machine processing may not be able to accurately identify some important entry data, so a manual identification and deletion method was chosen. The main operation was to divide the participating team members into three groups, who identified and censored the pandemic-related hot searches in turn while repeatedly checking and correcting them until a consensus was reached to confirm the entries. As there were no hot search terms related to the pandemic during the 10 to 14 January and 16 to 19 January periods, the sample did not include data from these two time spans. In total, 822 pandemic-related hot searches were finally retrieved after data cleaning.

Public attention provides a crucial basis for selecting hot search commentary samples. The pandemic-related hot searches retrieved by the team contained information, such as entry time, entry content, and entry ranking, etc. An event’s ranking in the hot search list indicates how much attention the event has received from the public. At the exact moment on a given day (noon), the higher the entry ranking, the higher the public’s attention was given to the entry content. Meanwhile, agency platforms with authoritative background are characterized by a large audience, diverse structures, high interactive participation, complete openness of comments, and rapid updates, the primary medium for discussions about online topics [50]. Therefore, based on the daily hot searches related to the pandemic at 12:00 h, this paper selected the highest-ranked hot searches at that moment and obtained the text from each commentary section after the trending event was reported by authoritative or influential platforms (e.g., “CCTV News” and “People’s Daily”). The corpus was used as research material, and texts for 35 days of commentaries were acquired, with a total of 7548 commentaries.

**Figure 1:**
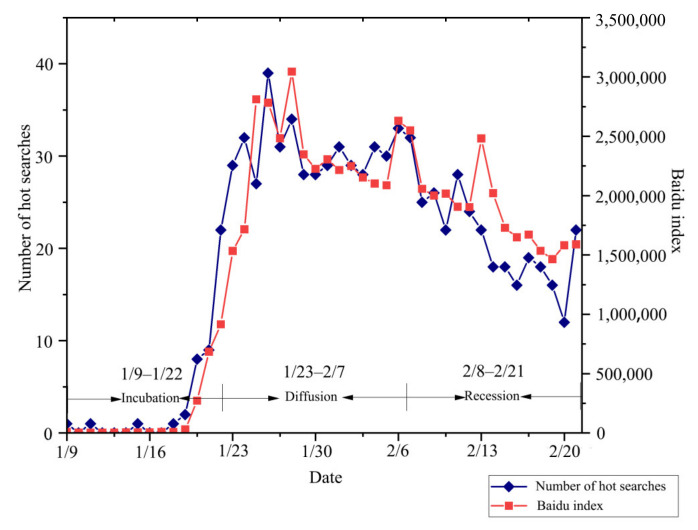
The changes of the number of Weibo hot searches and the Baidu index. (9 January 2020 to 21 February 2020).

Weibo hot search terms are keywords or topics that are frequently searched or discussed by people at any particular time. The larger the number of hot entries related to the pandemic, the higher the attention the public paid to the event. On this basis, it is feasible to categorize the stages of change in public attention by keeping track of variations in the numbers of hot searches relating to the pandemic during the study period. Given the limitations of single-source hot search samples, this paper also used another common index of the public attention to certain events, the Baidu index [51,52,53], to corroborate the classification results. Jieba word classification database tools were used to process the content of the hot search entries, with “novel coronavirus”, “pandemic”, “case”, “pneumonia”, and “new pneumonia” being identified as the top five keywords. In the end, the entries with the highest frequency (“novel coronavirus” and “pneumonia”) had already been included in the Baidu indices and were selected as the most representative Baidu search terms to be superimposed to obtain the comprehensive value of the Baidu index. The change in the number of Weibo hot searches and the integrated value of the Baidu index were combined as presented in Figure 1, and showed a shared pattern of shift where the initial value was almost zero, before increasing sharply and swiftly, and subsequently exhibiting a slightly fluctuating, continuous decrease.

After cross-reference with the data obtained from network public opinion monitoring software, it was observed that this pattern of change conformed to the three-stage model of network public opinion dissemination [54]. Accordingly, changes in group attention can be roughly divided into three stages, as shown in Figure 1. The incubation period ran from 9 January to 22 January 2020, and during this period, the group was less attentive. A significant increase in group attention occurred during the diffusion period between 23 January and 7 February. The period between 8 February and 21 February saw a less marked fluctuation in group attention.

### 3.3. Methods and Tools

#### 3.3.1. Text Review Sentiment Analysis

Textual review is a crucial channel for conveying emotion [55]. Using sentiment analysis tools to sift text, it is possible to derive a corpus containing three types of sentiment—positive, negative, and neutral—that captures the group’s sentiment tendencies and provides an adequate basis for analyzing the collective resilience of the community. Thematic clustering and textual commentary analysis are then conducted to examine emotional characteristics and any evolving sentiment. A text-mining tool known as ROST CM 6.0 supports sentiment analysis and is widely used in management, intelligence, geography and other research fields [56,57,58]. Once the custom and deactivated word lists have been set, the number of positive, negative, and neutral textual commentaries daily can be identified. They are then assigned a value of 1 for positive, −1 for negative, and 0 for neutral. After that, all the valued polarity category entries in the daily text commentaries are added up to obtain the text sentiment value sum of these daily commentaries, before being averaged to the text sentiment value for each day.

#### 3.3.2. Grounded Theory and NVivo 11

Grounded theory is a qualitative research method that emphasizes theoretical specificity and contextuality. Its core steps include open, axial, and selective coding at three levels. The coding process involves the gradual conceptualization, categorization, and theorizing of the source material [59]. The main tool for implementing the coding process of grounded theory is NVivo 11, which is a highly efficient qualitative research tool with powerful coding functions that can improve coding efficiency and effectively shorten the research cycle. Grounded theory and NVivo 11 were used to construct a psycho-emotional model to explore further the characteristics and influencing factors of collective resilience.

## 4. Results

Thematic and factor-process analyses based on temporal change are the main contents of collective resilience research from a psychological perspective [60]. After the outbreak of COVID-19, social groups exhibited a strong desire to obtain timely and effective information based on their sensitivity to life, fear of uncertainty and anxiety about being uninformed [61], During this time, collective resilience and emotions exhibited staged characteristics. In this way, grounded theory was therefore used to further identify any factors influencing collective resilience from a psycho-emotional perspective.

### 4.1. Thematic Evolution and Emotional Change

#### 4.1.1. Characteristics of Themes and Collective Resilience

Revealing any trends in the evolution of public opinion theme evolution is the basis for identifying the characteristics of collective resilience at different stages of a crisis. The reference points with similar meanings were combined into sub-themes, and then further summarized into themes, by reading the entries and summarizing their thematic meaning, as shown in Table 1.

**Figure 2:**
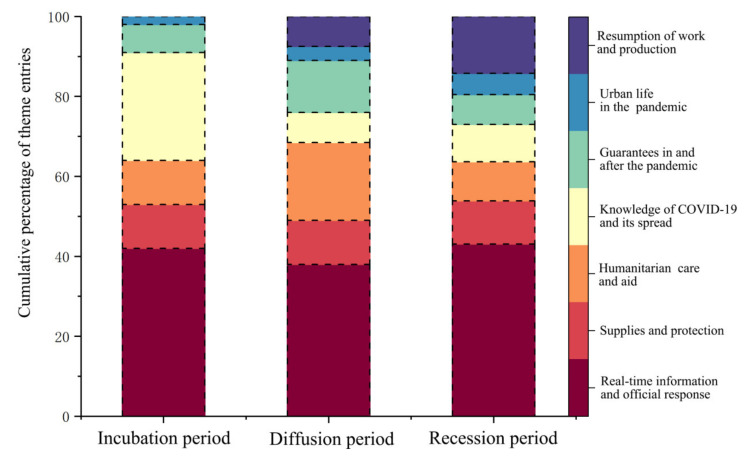
Thematic evolution with percentage in three stages.

Theme 1 includes reports on the situation of the pandemic in provinces and cities across the country, the risk of infection, and the clarification of rumors. Theme 2 covers supplies and protection. Theme 3 includes the humanitarian care and the assistance provided by multiple organizations. Theme 4 is about knowledge of the virus properties and its spread. Theme 5 covers the protection measures implemented by government in response to the pandemic. Theme 6 reflects the state of the city and its citizens during the pandemic. Theme 7 involves the resumption of work and production. Taking the temporal stage to which the themes belong as the horizontal axis and the percentage of each theme as the vertical axis, the number of entries of each theme changes with time, as shown in Figure 2.

“Real-time information and official response” was the most popular theme in all three phases of China’s first COVID-19 outbreak and control period. Live broadcasts of the pandemic situation in provinces and cities across the country, as well as authoritative interpretations and responses, enabled the people to understand the situation in the country in a timely manner. The central government was able to influence public opinion and regulate social sentiment to some extent, and this was the cornerstone of the positive representation of collective resilience from a psycho-emotional perspective [62]. The themes with the second highest proportion in the three phases of incubation, diffusion and recession were, respectively: “Knowledge of virus and COVID-19”, “Humanitarian care and aid”, and “Resumption of work and production”. As the pandemic evolved, prevention and control measures also continued to be adapted and implemented. Social groups have transitioned from requiring simple and factual pandemic information in the early stages to needing detailed information about affected areas in the mid-term, and then to requiring information regarding the resumption of work and production during the outbreak’s later stage. A time series three-stage model of collective resilience to accommodate the evolution of group information needs is therefore proposed in this study. The incubation period is characterized by preparatory resilience, when frontline healthcare workers and medical experts carried out treatment and virus research as well as disseminating their knowledge so that the public could quickly understand the characteristics of the virus and prepare themselves for the effective fight against the pandemic in its middle and late stages. During the diffusion period, society took humanitarian care and assistance as led by the government with the participation of institutional groups such as medical and nursing staff and enterprises, reflecting the resilience of the process. During the recession period, the pandemic was largely contained, and the themes of when and how to resume work rose in popularity. Social groups needed to adapt to the socio-economic and cultural changes brought about by the pandemic and faced diverse and developing livelihood options or crises. From the perspectives of enhancing collective physiological coping capacity and social adaptability, respectively, the government put forward measures such as “free vaccination” and “employment assurance” in an effort to enhance collective resilience. The groups’ recovery has proven to be resilient.

#### 4.1.2. Characteristics of Emotional Changes

**Figure 3:**
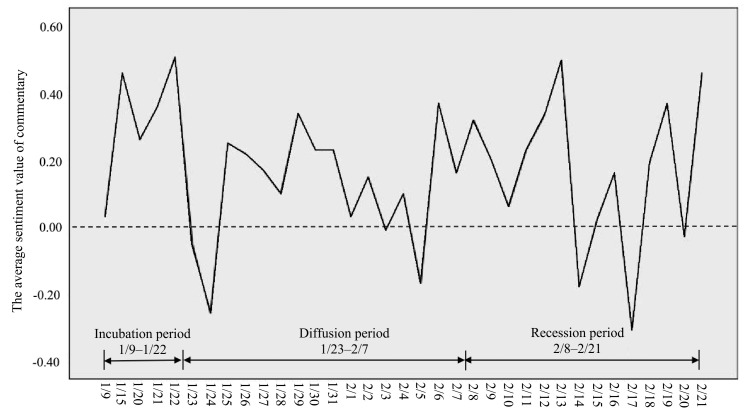
Sentiment Value Change of Weibo Hot Search Commentaries.

Group sentiment is the most intuitive variable reflecting the psychological dimension of collective resilience. After obtaining the commentary text of relevant hot search topics, this study conducted manual denoising measures such as the removal of duplicate commentaries or those that were commercial in nature, as well as comments without sentiment characteristics and meaning, to ensure the authenticity of the sampling. Finally, 5492 out of the original 7548 commentaries were obtained and saved in a uniform txt format that ROST CM 6.0 can recognize. After the data processing was completed, the sentiment analysis function panel in ROST CM 6.0 was used to present positive, negative and neutral sentiment values for all commentaries and to access the overall judgment of sentiment tendency, as shown in Figure 3.

Group emotion showed fluctuations during China’s first anti-pandemic cycle. Generally, group emotion was predominantly positive, with negative sentiments prevalently occurring during the diffusion and recession periods. In terms of stages, the domestic pandemic did not show its severity during the incubation period, and the mean sentiment value was positive. During the diffusion period, the overall sentiment value remained positive, but there were significant negative abnormalities. Throughout the country, the number of confirmed cases and deaths increased after 23 January. Following that, the negative information proliferated dramatically, including rumors, news that merchants had raised prices, or claims that anti-pandemic supplies were full of counterfeit and inferior products, triggering a significant loss in sentiment value. Upon controlling the pandemic situation on February 8, positive news focused on the “national pandemic recovery map”; “many provinces and cities started to resume work and production”; and “the government’s appropriate tax and social security reductions and exemptions for enterprises” became the focus of news coverage, and the initial positive sentiment value was gradually restored.

It is noteworthy that negative sentiment value outliers appeared seven times during the study period, corresponding to the different events that occurred on that day; these were categorized into five types, as shown in Table 2.

As knowledge about the spread and transmissibility of the virus became widely known, there was a growing fear that new transmissible cases would arise. After the discovery of two unexplained cases in Xinyang, Henan Province, commentaries stated that “the transmission route was complicated and it is difficult to analyze and screen the source of infection” and “the virus is too scary and cunning”. The sentiment value of the groups reached a low of −0.26 on 24 January when a Hubei family of three was reported to have gone to Shandong for Chinese New Year. Those who concealed facts, intentionally infected others, or increased their risk of infection aroused the group’s anger, causing them to make comments such as: “Do not go around harming others and yourself.” It was disappointing that masks donated by Malaysian compatriots were mistaken for bombs, causing negative emotions among the community, a community that was already suffering a shortage of anti-pandemic materials; commentaries included, “masks are too painful and too wasteful”, recorded on 3 February. During the recession period, people focused on their rights and interests during the process of resuming work and production. Opinions like “the delayed start of school can be made up for in the summer”, which represented not-entirely-reasonable work requirements, triggered the dissatisfaction among teachers, who helplessly sighed, “The daily live teaching is frustrating”.

On 20 February, a news report entitled “The number of Wuhan’s new confirmed cases is higher than that of Hubei Province” hit the hot search top spot, and the concepts of “new addition” and “verified reduction” were questioned. Most people demanded that “there should be no number games, and the details of both new additions and verified reductions should be made clear”.

The government and the public played major roles in the prevention and control of the pandemic, with the public being the main beneficiary of the government’s effective implementation of anti-virus measures. The government’s timely disclosure of accurate factual information, the protection of the legitimate rights and interests of groups in the midst of the pandemic, and the active cooperation of citizens as well as their participation in pandemic prevention and rescue operations, all effectively alleviated the group’s negative psychology in the face of this crisis.

### 4.2. Influencing Factors of Collective Resilience 

#### 4.2.1. Building a Model using Grounded Theory

Text-review analysis based on grounded theory is an essential method that can be used to reveal the factors influencing collective resilience from a psycho-emotional perspective. Three copies of the review text were reserved in accordance with the “information saturation principle” prior to the analysis, and the results were checked for saturation using the text that was reserved. Considering the differences in the phases of the theme, one text was randomly selected from each phase as a reserved text according to the previous division of the phases (Figure 2), for a total of three reserved texts. The rest of the cleaned text data were imported sequentially into the NVivo 11 tool, and the collected daily commentaries were named sequentially as S* (e.g., S1 for the text of 9 January). Through repeated recitation and comprehension of the corpus sentence by sentence, the individual phenomena in each sentence, such as “believe in Wuhan” and “love hometown” were labeled. Labels with similar or repeated meanings were summarized to obtain thirty-one concepts, such as place attachment (Table 3).

Concepts with the same attributes were then clustered and refined to form seven initial categories (Table 4). The axis coding discovered and established logical connections between initial categories and reorganized them into higher-level categories according to their relevance [63], resulting in four main categories: antecedent factors, explicit factors, implicit factors and emotional tendency.

Selective coding further distilled the core categories, linked them with other categories, and described the behavioral phenomena and the context through a “story line” to build conceptual frameworks and develop new theories (Table 5). The research found that “factors and mechanisms influencing collective resilience from a psycho-emotional perspective” can be a core category used to unify other categories. The story line was that a progressive relationship existed among the antecedent factors, explicit factors and implicit factors, with all three acting in combination and influencing the emotional disposition of the group. The 35 texts used in this step were verified as covering all the theme types in Table 1. The sample size of the commentaries was sufficient to study a specific topic, so the results were reliable [64]. Finally, a saturation test was conducted, and the study proved that the constructed-saturation test of the grounded theory passed. The theoretical model was saturated by recoding the data from the three commentary samples set aside, and no new concepts, categories or relationships emerged.

#### 4.2.2. Interpretation

(1) Basic cognition

Basic cognition includes three concepts: environmental risk perception, national identity, and place attachment. Research has suggested that the higher the level of risk perception, the stronger the public response to an the event [8]. Conversely, the more peaceful the public are, the less pronounced any negative emotions stemming from the event may be. Meanwhile, Protection Motivation Theory is used to interpret the relationships between, and roles played by, risk perception and coping strategies [65]. As part of its perceived risk assessment process, the group was sensitive to the pandemic environment and their distance from the risk, as well as the infectiousness and severity of the disease. With the continuous reporting of pandemic news, social groups gradually accepted the reality and adapted to the social environment resulting from the outbreak. Moreover, there were no confirmed cases reported in most provinces and cities initially, and overall, the situation remained stable. As a result, the level of public risk perception level decreased rapidly at this stage. In addition, it was stated that “many people did not know they are in a pandemic area”. The public generally perceived themselves as distant from the disease, which reduced any infection risk assessment [8]. Furthermore, the group deemed COVID-19 to be less dangerous than SARS. Therefore, people made commentaries such as “it’s not SARS and it’s not highly contagious. There is no need to panic and everyone can just go calmly home for the New Year.””. Inaccurate assessments of the virus’ infectiousness and severity therefore affected people’s perception of risk. At the same time, the public exhibited a strong dependence on, and attachment to, local and national governmental actions. Some netizens commented that, “We believe in the country’s ability to adapt”, and others stated that, “Although Wuhan may not be perfect, we believe in it.” Consequently, the groups could maintain a certain level of resilience as they perceived the crisis environment as low risk with their attachment to the place and national identity as antecedent factors.

(2) Intermediary condition

**Figure 4:**
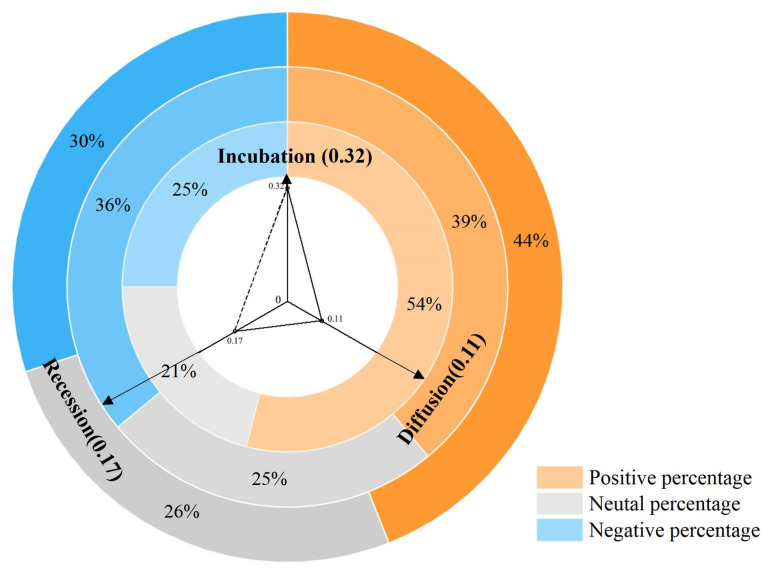
Percentage of emotional polarity and the average sentiment value by stage.

Intermediary conditions include explicit factors such as government and corporation behavior, and implicit factors such as institutional strengths, coordination of collective resource allocation, spiritual identity, collective memory, etc., all of which can influence collective resilience from a psycho-emotional perspective. According to the calculation, the sentiment value of the incubation period (9 January–22 January 2020) was 0.32, with a high percentage of positive sentiment in the group of 54%, as shown in Figure 4.

On the one hand, the central government interpreted the situation and guided social groups on prevention and control through social media platforms. On the other hand, local governments had begun to initiate emergency responses to major public health emergencies. As part of a the national appeal, public awareness of the pandemicimproved, and self-protection measures were largely adopted. This is evidenced by the group’s commentaries such as, “we must wear masks” and “we need to improve our own immunity”.

During the diffusion period (23 January–7 February 2020), the sentiment value dropped to 0.11, and the negative sentiment percentage was 36%. The reasons for this change were the multiple factors involved in road-traffic control, the increase in infected people, and a shortage of medical supplies. In their efforts to combat the pandemic, various organizations played to their strengths. The Central Government, the Central Committee of Communist Party of China (CPC), and the Ministry of Foreign Affairs all played important roles in the anti-pandemic strategy. On 23 January, the Central Government effectively locked the city of Wuhan down to control the export of pathogens. On 30 January, the National Healthcare Security Administration issued the “Notice on Medical Coverage of COVID-19”, preliminarily solving the problem of medical expenses incurred by patients. On 31 January, the Ministry of Foreign Affairs stated that the Chinese government had decided to send civil aviation charter flights abroad to bring home Hubei citizens stranded overseas. As the pandemic worsened in China and continued to spread globally, the government implemented a series of initiatives to safeguard people’s lives, reflecting the strengths of the country’s people-centered, democratic and centralized institutional framework. In resource terms, new medical treatment centers were built by the government. Corporations, social organizations and the general public actively participated in the battle against the pandemic, such as the Air Force’s emergency transportation of 10,000 tons of equipment and materials, corporations’ willing production of additional supplies, social organizations’ donations of supplies, and people’s participation in volunteer teams, etc. The efficient dispatch and usage of collective resources such as personnel and materials, as well as the correct perception of the pandemic, provided a strong basis for curbing the spread of the pandemic. Moreover, the community’s recognition of the deeds of heroic figures such as Nanshan Zhong, a famous respiratory pathologist who made a great contribution to the fight against COVID-19 in Wuhan, reinforced the collective memory of the event of “fighting COVID-19 in 2020”. Based on the identification of the anti-pandemic spirit and culture, the collectivist value orientation of social groups formed. Under the influence of the behavior of each subject, and their behavioral characteristics and spiritual connotations, the sentiment value of the group decreased significantly at this stage but remained positive, and the psychological resilience of the group also strengthened.

During the recession period, group sentiment value recovered to 0.17 which is higher than diffusion but lower than incubation, the positive emotions percentage was 43%. The public switched their attention from Wuhan City, Hubei Province to the hardest hit area of the pandemic, to Zhejiang Province, which was leading China’s economic recovery. The focus of commentaries changed from “rescue” to “production”, “resumption of work”, and “online class”. At this stage, the accelerated recovery of group psychology depended first and foremost on the issuance and understanding of relevant regulations for the resumption of work and production as well as the distribution of free vaccines, thus ensuring at a governmental level the safety of social group and an ability to manage their lives within the context of the difficult conditions necessitated by the pandemic. The second factor was the collaboration between social organizations and government departments, as well as the full use of public initiatives. These organizations helped maintain an effective pandemic-control program and assisted in areas suffering severely from the disease. Continued vigilance and the mask-wearing requirement were supported. In addition, the collective sense of identification within the region, such as “ the spirit and style of Zhejiang”, the collective memory of special landscapes, such as “the Square Cabin Hospital” and an identification with heroic figures such as “Yong Ying” (Mayor of Shanghai in 2017, secretary of the CPC Hubei Provincial Committee at a critical time in 2020 and a great contributor to the fight against COVID-19 in Wuhan.). It had become crucial support for groups to seek social identity and a sense of belonging and dependence [66,67,68]. The orientation of collective values further strengthened during this period, with the group showing positive sentiment and optimistic responses, enhancing its psychological resilience.

**Figure 5:**
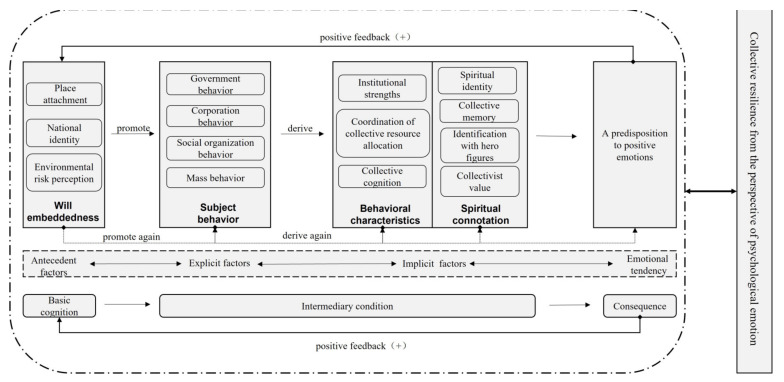
A model of factors influencing collective resilience from a psycho-emotional perspective.

In conclusion, a model of factors influencing collective resilience from a psycho-emotional perspective was constructed, as shown in Figure 5. The positive feedback framework of “basic cognition-intermediary condition-consequence” explained the process of, and pattern in, collective resilience. The storyline was as follows: in times of crisis, the group turned to social managers for assistance. Driven by the core concept of “people-centered” governance, the government started initiated governmental actions with the participation of multiple agents. All subjects joined the anti-pandemic rescue action plan. A sense of community and common crisis awareness in society was engendered by the behavioral characteristics (institutional strengths, coordination of collective resource allocation, collective cognition, etc.) and spiritual connotation (spiritual identity, collective memory, identification with hero figures, and collectivist values, etc.). In response to multiple factors, the group showed positive sentiments during the crisis caused by the pandemic. The feedback mechanism of social group psychological resilience promotes the formation of positive group sentiment, which in turn strengthens the group’s sense of place attachment, national identity, and environmental risk perception in the post-crisis era.

## 5. Discussion

Human survival and development are in grave danger as a result of COVID-19, the most devastating infectious disease outbreak in a century. To cope with external shocks, social groups have united to function as a collective resource. Collective resilience in the psychological dimension affects the time groups may take to recover and adapt after the depression caused by crises such as the recent pandemic. The rapid psychological adaptation and effective reconstruction of social groups can play a critical role in dealing with sudden public crises such as COVID-19. Emotion is a direct expression of the psychological resilience required to adapt flexibly and recover effectively from a crisis, so it has become a significant method for quantifying collective resilience from a psychological dimension. To make social groups more resilient and social psychological reconstruction more efficient is essential when dealing with public crises. Empirical research based on COVID-19 could also provide a realistic basis for building collective resilience during the emergency prevention of future public crises. This research aims to promote the building of collective resilience from a psycho-emotional perspective to help cope with any challenges and risks arising. It provides fresh advantages for fostering social stability and a cohesive populace, as well as tactical advantages for the sustainability of the country and region. Owing to the lack of a systematic theoretical research framework of collective resilience factors, the exploratory grounded theory research method was introduced into the process of exploring the factors influencing collective resilience in order to analyze the process of events, which are not easily perceived by people during the pandemic. Bottom-up analysis and generalization were demonstrated to be able to lead gradually to a theoretical model of collective resilience which could conform to the objective reality, and therefore has application value.

## 6. Conclusions

During the first anti-pandemic cycle in China, the initial plan was to provide diverse and differentiated information to people in a phased manner. As the pandemic progressed, collective resilience displayed could be characterized as exhibiting a “preparation–process–recovery” pattern. During the incubation period, groups demonstrated preparation resilience. The government dedicated itself to scientific research, sending more medical experts to the frontline as well as disseminating pandemic information and knowledge to the public through internet and social networking platforms to reduce the risk of the virus spreading. The group showed process resilience during the diffusion period. Multiple subjects participated in the production and deployment of medical materials to meet the demand for supplies. During the recession period, while promoting the resumption of work and production, the government made institutional guarantees such as the provision of vaccination subsidies and corporate tax incentives to enhance the physiological adaptability of groups and ensure their livelihoods, reflected in the group’s recovery resilience.

A regular pattern of change in group emotions was observed. The sentiment of the group tended to be positive in general, and a “positive emotion prevailing—negative emotion appearing—positive emotion recovering” trend was observed. Negative emotional values emerged in close association with governmental and public behavioral events. Satisfying the collective’s information needs and accurately communicating a sense of risk and responsibility, along with ensuring that the rights and interests of the collective were protected, was a practical way to decrease the likelihood that people would feel negative about the crisis.

Positive feedback revealed the psycho-emotional factors influencing of collective resilience based on the “basic cognition–intermediary condition–consequence” theory. The basic cognition of a group is place attachment, national identity, and environmental risk perception when a crisis strikes. The action of subjects such as the government, corporations, social organizations, and the public, as well as their behavioral characteristics such as institutional strengths, coordination of collective resource allocation, collective cognition, and the resulting spiritual connotations such as spiritual identity, collective memory, identification with hero figures, and collectivist value, was the intermediary conditions that influenced the psychological dimension of collective resilience.

## Figures and Tables

**Table 1:** Hot Search Themes Clustering.

Serial Number	Themes	Sub-Themes	Reference Points
1	Real-time information and official response	Real-time situation of national outbreak by province and city; Coverage of journeys with risk of infection; Refutation of rumors	“National outbreak maps”; “Looking for passengers who have taken the D3286 train”; “Do not rush to buy and self-medicate with *Dual Yellow Lian Oral Liquid*”
2	Supplies and protection	Epidemiological hospital construction and commissioning; Production and delivery of anti-pandemic materials such as masks; Physical, chemical, and biotechnological protection measures	“Wuhan has built anti-pandemic hospital using the Xiaotangshan Model” (Xiaotangshan Model: The Xiaotangshan Hospital was a temporary emergency place built rapidly in 2003 in response to the outbreak of SARS in Beijing. The model has now become a form of prevention and treatment for cities in China to respond to the increased risk of potential epidemics by concentrating superior resources on the centralized, specialized treatment and management of patients in order to effectively treat them and contain the pandemic at its source); “The Ministry of Commerce deployed over 2 million masks”; “The city of Hangzhou shut down public places not providing life necessities”; “Wuhan conducted citywide disinfection and sterilization action”; “Special immune plasma products COVID-19 put into clinic use”
3	Humanitarian care and aid	Rescue operations involving the medical and nursing community, social organizations, enterprises, and ordinary people	“Doctors from Beijing Unions Medical College Hospital volunteer to support”; “Wenchuan villagers spontaneously provide Wuhan with 100 tons of vegetables”; “Academician Qian Qihu donates 6.5 million to Wuhan”; “Enterprise from Henan Provinces have developed new types of quarantine cap”
4	Knowledge of COVID-19 and its spread	The targets and ways of pandemic spreading; Interpretation of the virus’ properties	“A novel coronavirus as the causative agent of unexplained pneumonia in Wuhan”; “Pneumonia does not rule out the possibility of limited human-to-human transmission”
5	Guarantees in and after the pandemic	Traffic control; Financial subsidies	“Unauthorized closure of highway entrances and exits is strictly prohibited”; “The State Council decides to temporarily exempt enterprises from social security payment”
6	Urban life in the pandemic	The state of urban people’s lives; The city state and condition	“Nurse encourages patient to play Tai Chi in the ward”; “Remote sensing image showing Wuhan’s differences before and after the outbreak”
7	Resumption of work and production	Postponement of various work activities; Time when, and ways to, resume work; How to resume work and production	“Delayed resumption of work in Zhejiang”; “Delayed work and school resumption in Hunan Province”

**Table 2:** Hot Search events corresponding to sentiment value outliers.

Type of Events	Subject	Time	Hot Search Events
A new type of transmissible event	Public-led	23 January	Six cases of family outbreaks were detected in Guangdong
		17 February	Two unexplained cases in Xinyang, Henan
Malicious concealment leading to the spread of the pandemic		24 January	A family of three from Hubei Province who went to Shandong for the Lunar New Year was reported
		5 February	Patient concealment led to the quarantine of 68 medical personnel
The misunderstanding of invalid donations		3 February	A suspicious package of explosion risk was found to be full of masks
Group rights-based event	Government-led	14 February	Many local governments clarify that delayed school opening times can be made up during the summer holidays
Event of information inaccuracy		20 February	Why is the number of Wuhan’s new confirmed cases higher than that of Hubei Province

**Table 3:** Opening coding (excerpts).

Original Representative Statement	Labels	Concepts	Initial Categories
(S1) Although Wuhan may not be perfect, we believe in it	Believe in Wuhan	Place attachment	Will embeddedness
(S3) I am in Nanjing and desperately want to go back to my hometown in the North East	Miss hometown and want to go home		
(S4) 40,000 families in Wuhan share neighborhood love	Neighborhood support in a pandemic		
(S6) My city is sick, but we will cure it. You are welcome to come back to Wuhan in the future	Love hometown		
(S1) Thanks to the national and expert groups	Gratitude to the country	National identity	
(S5) We all need to believe in our country	believe in the country		
(S9) The central government is wise to take over Wuhan	Praise national decisions		
(S14) No matter when, the motherland will never abandon any Chinese person	the country’s single-mindedness focus on the people		
(S1) it’s not SARS and it’s not highly contagious. There is no need to panic and everyone can just go calmly home for the New Year.	Viruses are not to be feared	Environmental risk perception	
(S3) Many people are also unaware that they are in an area impacted by COVID-19	No awareness of the pandemic		

**Table 4:** Axial coding.

	Main Categories -Axial Coding	Initial Categories-Open Coding	Concepts	Connotation
Basic cognition	Antecedent factors	Will embeddedness	Place attachment	The group assessed risk via environmental risk perception in the early stage of the pandemic. The group showed trust in and dependence on the local and national government, then formed a common group will.
			National identity	
			Environmental risk perception	
Intermediary condition	Explicit factors	Subject behavior	Government behavior	Behaviors of various subjects in the process of fighting the COVID-19 pandemic.
			Corporation behavior	
			Social organization behavior	
			Mass behavior	
	Implicit factors	Behavioral characteristics	Institutional strengths	Behavioral characteristics of various subjects.
			Coordination of collective resource allocation	
			Collective cognition	
		Spiritual connotation	Spiritual identity	The group had a special memory and sense of identity with the heroic figures, special landscapes, and spirits associated with the anti-pandemic activities, then formed collectivist value.
			Collective memory	
			Identification with hero figures	
			Collectivist value	
Consequence	Emotional tendency	Positive emotion	Appreciation; Love; Hope; Effort; Support; Enron	As the pandemic progressed, groups showed different emotional responses.
		Negative emotion	Trepidation; Fear; Anxiety; Anger; Embarrassment; Regret	
		Neutral emotion	Prayer; Welcome; Auspice; Inspiration; Gratitude	

**Table 5:** Selective coding.

Typical Relationship	Type of Relationship	Structure of the Relationship
Antecedent factors → Explicit factors/Implicit factors	Progressive relationship	During the pandemic (on the basis of the group’s subjective will and risk perception as the basic cognition, the behavior of the government, corporations, and other subjects), the behavioral characteristics and spiritual connotations acted as intermediary conditions that jointly influenced the group’s emotional tendency.
Antecedent factors + Explicit factors/Implicit factors → Emotional tendency	Causality	

## Data Availability

Not applicable.

## References

[1] Leach M. (2008). Re-Framing Resilience: A Symposium Report.

[2] Pendall R., Foster K.A., Cowell M. (2010). Resilience and regions: Building understanding of the metaphor. Camb. J. Reg. Econ. Soc..

[3] Williams R., Drury J. (2009). Psychosocial resilience and its influence on managing mass emergencies and disasters. Psychiatry.

[4] Drury J., Cocking C., Reicher S. (2009). The nature of collective resilience: Survivor reactions to the 2005 London bombings. Int. J. Mass Emergencies Disasters.

[5] Meng B., Fang D.P., Li N. (2018). A literature review and theoretical framework of crowd resilience to disasters. China Saf. Sci. J..

[6] Manyena S.B., O’Brien G., O’Keefe P., Rose J. (2011). Disaster resilience: A bounce back or bounce forward ability?. Local Environ..

[7] Fredrickson B.L., Fowler R.D. (2001). The role of positive emotions in positive psychology: The broaden-and-build theory of positive emotions. Am. Psychol..

[8] Roeser S. (2012). Risk Communication, Public engagement, and climate change: A role for emotions. Risk Anal..

[9] Ferrer R.A., Klein W.M.P., Avishai A., Jones K., Villegas M., Sheeran P. (2018). When does risk perception predict protection motivation for health threats? A person-by-situation analysis. PLoS ONE.

[10] Mackie D.M., Devos T., Smith E.R. (2000). Intergroup emotions: Explaining offensive action tendencies in an intergroup context. J. Personal. Soc. Psychol..

[11] Martin-Breen P., Anderies J.M. (2011). Resilience: A literature Review.

[12] Masten A.S. (2001). Ordinary magic: Resilience processes in development. Am. Psychol..

[13] Rhoads D.J. (1994). Resiliency research: An exploration of successful coping patterns. Eta Sigma Gamma Monogr. Ser..

[14] Flach F.F. (1988). Resilience: Discovering a New Strength at Times of Stress.

[15] Ntontis E., Drury J., Amlôt R., Rubin G.J., Williams R. (2018). Emergent social identities in a flood: Implications for community psychosocial resilience. J. Community Appl. Soc. Psychol..

[16] Aslani F., Amini H.K. (2022). Evaluation of the impacts of identity and collective memory on social resilience at neighborhood level using grounded theory. Space Cult..

[17] Prestes C.R.S., Paiva V.S.F. (2016). Psychosocial approach and health of black women: Vulnerabilities, rights and resilience. Saúde E Soc..

[18] Tippens J.A., Roselius K., Padasas I., Khalaf G., Kohel K., Mollard E., Sheikh I. (2021). Cultural bereavement and resilience in refugee resettlement: A photovoice study with Yazidi women in the midwest United States. Qual. Health Res..

[19] Drury J., Williams R. (2012). Children and young people who are refugees, internally displaced persons or survivors or perpetrators of war, mass violence and terrorism. Curr. Opin. Psychiatry.

[20] Cheng H., Kim H.Y., Tsong Y., Joel Wong Y. (2021). COVID-19 anti-Asian racism: A tripartite model of collective psychosocial resilience. Am. Psychol..

[21] Seger C.R., Smith E.R., Mackie D.M. (2009). Subtle activation of a social categorization triggers group-level emotions. J. Exp. Soc. Psychol..

[22] Parkinson B. (2020). Intragroup emotion convergence: Beyond contagion and social appraisal. Personal. Soc. Psychol. Rev..

[23] Lei S. (2020). Research and analysis of cross-cultural communication of social media in Southeast Asian countries: The collective identity of social culture. Front. Educ. Res..

[24] Garcia D., Rimé B. (2019). Collective emotions and social resilience in the digital traces after a terrorist attack. Psychol. Sci..

[25] Han X., Wang J., Zhang M., Wang X. (2020). Using social media to mine and analyze public opinion related to COVID-19 in China. Int. J. Environ. Res. Public Health.

[26] Wang J.L., Zhang M., Han X.H., Wang X., Zheng L. (2020). Spatio-temporal evolution and regional differences of the public opinion on the prevention and control of COVID-19 epidemic in China. Acta Geograohica Sin..

[27] Dosono B., Semaan B. (2020). Decolonizing tactics as collective resilience: Identity work of AAPI communities on Reddit. Proc. ACM Hum. -Comput. Interact..

[28] Valizadeh N., Ghazani E., Akbari M., Shekarkhah J. (2022). How do collective efficiency and norms influence the social resilience of Iranian villagers against the COVID-19? The mediating role of social leadership. Front. Public Health.

[29] Louis E.F., Eugene D., Ingabire W.C., Isano S., Blanc J. (2022). Rwanda’s resiliency during the Coronavirus Disease Pandemic. Front. Psychiatry.

[30] Bentley J.A., Mohamed F., Feeny N., Ahmed L.B., Musa K., Tubeec A.M., Angula D., Egeh M.H., Zoellner L. (2020). Local to global: Somali perspectives on faith, community, and resilience in response to COVID-19. Psychol. Trauma-Theory Res. Pract. Policy.

[31] Saghin D., Lupchian M.M., Luches D. (2022). Social cohesion and community resilience during the COVID-19 pandemic in Northern Romania. Int. J. Environ. Res. Public Health.

[32] Parenteau A.M., Boyer C.J., Campos L.J., Carranza A.F., Deer L.K., Hartman D.T., Bidwell J.T., Hostinar C.E. (2022). A review of mental health disparities during COVID-19: Evidence, mechanisms, and policy recommendations for promoting societal resilience. Dev. Psychopathol..

[33] Silveira S., Hecht M., Matthaeus H., Adli M., Voelkle M.C., Singer T. (2022). Coping with the COVID-19 pandemic: Perceived changes in psychological vulnerability, resilience and social cohesion before, during and after lockdown. Int. J. Environ. Res. Public Health.

[34] Jiang J., Liu Y., Han P., Zhang P., Shao H., Peng H., Duan X. (2022). Psychological resilience of emergency nurses during COVID-19 epidemic in Shanghai: A qualitative study. Front. Public Health.

[35] Olcoń K., Allan J., Fox M., Everingham R., Pai P., Keevers L., Mackay M., Degeling C., Cutmore S.-A., Finlay S. (2022). A narrative inquiry into the practices of healthcare workers’ wellness program: The SEED experience in New South Wales, Australia. Int. J. Environ. Res. Public Health.

[36] Plessas A., Paisi M., Baines R., Wheat H., Delgado M.B., Mills I., Witton R. (2021). Frontline experiences and perceptions of urgent dental care centre staff in England during the COVID-19 pandemic: A qualitative study. Br. Dent. J..

[37] Israelashvili J. (2021). More positive emotions during the COVID-19 pandemic are associated with better resilience, especially for those experiencing more negative emotions. Front. Psychol..

[38] Alhothali G.T., Al-Dajani H. (2022). Emotions and resilience in Saudi women’s digital entrepreneurship during the COVID-19 pandemic. Sustainability.

[39] Yang D., Swekwi U., Tu C.C., Dai X. (2020). Psychological effects of the COVID-19 pandemic on Wuhan’s high school students. Child. Youth Serv. Rev..

[40] Venkatesh H., Osorno A.M., Boehm J.K., Jenkins B.N. (2022). Resilience factors during the Coronavirus pandemic: Testing the main effect and stress buffering models of optimism and positive affect with mental and physical health. J. Health Psychol..

[41] Guèvremont A., Boivin C., Durif F., Jenkins B.N. (2022). Positive behavioral change during the COVID-19 crisis: The role of optimism and collective resilience. J. Consum. Behav..

[42] Marzouki Y., Aldossari F.S., Veltri G.A. (2021). Understanding the buffering effect of social media use on anxiety during the COVID-19 pandemic lockdown. Humanit. Soc. Sci. Commun..

[43] Zhang X., Zhou Y., Zhou F., Pratap S. (2021). Internet public opinion dissemination mechanism of COVID-19: Evidence from the Shuanghuanglian event. Data Technol. Appl..

[44] Li J., Tang X., Dong D. (2021). Identification of public opinion on COVID-19 in microblogs. Proceedings of the 16th International Conference on Computer Science & Education (ICCSE).

[45] Xiao Q., Huang W., Zhang X., Wan S., Li X. (2021). Toward Internet rumors during the COVID-19 pandemic: Dynamics of topics and public psychologies. Front. Public Health.

[46] Zhong Z. (2021). Internet public opinion evolution in the COVID-19 event and coping strategies. Disaster Med. Public Health Prep..

[47] Yu W., Chen N., Chen J. (2022). Characterizing Chinese online public opinions towards the COVID-19 recovery policy. Electron. Libr..

[48] Chiang Y., Chu M., Lin S., Cai X., Chen Q., Wang H., Li A., Rui J., Zhang X., Xie F. (2021). Capturing the trajectory of psychological status and analyzing online public reactions during the Coronavirus Disease 2019 pandemic through Weibo Posts in China. Front. Psychol..

[49] Li L.F., Zhang Q.P., Wang X., Zhang J., Wang T., Gao T.-L., Duan W., Tsoi K.K., Wang F.-Y. (2020). Characterizing the propagation of situational information in social media during covid-19 epidemic: A case study on Weibo. IEEE Trans. Comput. Soc. Syst..

[50] Zhao J.D., Shan Q. (2017). Research on microblog agenda setting of multiple participants under emergency crisis events: A case study of “Dongfang Star” shipwreck. E-Government.

[51] Gong X., Han Y.Y., Hou M.C., Guo R. (2021). Online public attention during the early days of the COVID-19 pandemic: Infoveillance study based on Baidu Index. JMIR Public Health Surveill..

[52] Gong X., Hou M.C., Han Y.Y., Liang H.L., Guo R. (2022). Application of the internet platform in monitoring Chinese public attention to the outbreak of COVID-19. Front. Public Health.

[53] Xie T.T., Tang T., Li J. (2020). An extensive search trends-based analysis of public attention on social media in the early outbreak of COVID-19 in China. Risk Manag. Healthc. Policy.

[54] Wang Y.Q., Wu R., Zheng J., Xue P.Y. (2022). Research on the public opinion guidance mechanism of major public health incidents. Front. Psychol..

[55] Wang X., Tang L., Zhang L.N., Zheng J. (2022). Initial stage of the COVID-19 pandemic: A perspective on health risk communications in the restaurant industry. Int. J. Environ. Res. Public Health.

[56] Chen H., Wang M., Zhang Z. (2022). Research on rural landscape preference based on TikTok short video content and user comments. Int. J. Environ. Res. Public Health.

[57] Su H.W., Feng S.H., Tan R.H. (2021). An analysis of tourism emotional portraits by web crawler-taking Guangxi red tourism as an example. Proceedings of the 13th International Conference on Intelligent Computation Technology and Automation (ICICTA).

[58] Liu Y.H., Lai L.P., Yuan J. (2020). Research on Zhanjiang’s leisure sports tourism development strategy in coastal recreational areas. J. Coast. Res..

[59] Seidel S., Urquhart C. (2013). On the emergence and forcing in information systems grounded theory studies: The case of Strauss and Corbin. J. Inf. Technol..

[60] Xu C., Zhao Z.C., Wen T.Z. (2017). Resilience—Conceptual analysis and reconstruction from multidisciplinary perspectives. J. Hum. Settl. West China.

[61] Qian D., Li O. (2020). The relationship between risk event involvement and risk perception during the COVID-19 outbreak in China. Appl. Psychol.-Health Well-Being.

[62] Chen Q., Min C., Zhang W., Wang G., Ma X., Evans R. (2020). Unpacking the black box: How to promote citizen engagement through government social media during the COVID-19 crisis. Comput. Hum. Behav..

[63] Zhang S., Li J., Wang W.T. (2017). Influence factors of users’ participation willingness is sharing economy: A case study of Wechat Q&A. Libr. Trib..

[64] Guest G., Bunce A., Johnson L. (2006). How many interviews are enough? an experiment with data saturation and variability. Field Methods.

[65] Mileti D.S., Darlington J.A.D.R. (1997). The role of searching in shaping reactions to earthquake risk information. Soc. Probl..

[66] Liao Z.J., Dai G.Q. (2020). Inheritance and dissemination of cultural collective memory: An analysis of a traditional festival. Sage Open.

[67] Cheng E.W., Yuen S. (2020). Memory in movement: Collective identity and memory contestation in Hong Kong’s Tian an men vigils. Mobilization.

[68] Qian J.X., Zhu H. (2014). Chinese urban migrants’ sense of place: Emotional attachment, identity formation, and place dependence in the city and community of Guangzhou. Asia Pac. Viewp..

